# HIF-1α facilitates glioma proliferation and invasion by activating pyroptosis signaling axis

**DOI:** 10.1186/s41016-024-00366-3

**Published:** 2024-05-11

**Authors:** Xin-Wei Wang, Hao Fu, Ya-Min Zhang

**Affiliations:** 1 The First Central Clinical School, Tianjin Medical University, Tianjin, 300142 China; 2 Department of General Medicine, Characteristic Medical Center of PAP, Tianjin, 300162 China

**Keywords:** Glial cells, Hypoxia-inducing factor 1α, SHG44 cells, Pyroptosis

## Abstract

**Background:**

HIF-1α is thought to be a novel regulator which contributes to carcinogenesis. However, the mechanism underlying the effect of HIF-1α in gliomas remains largely unknown.

**Methods:**

In the research, we demonstrate that HIF-lα mRNA and protein levels are elevated in glioma cells. The colony formation assays, transwell assays, and wound-healing assays showed that overexpression of HIF-1α promoted proliferation and invasion of glioma cells.

**Results:**

Overexpression of HIF-lα also increased the expression of inflammatory factors related to pyrolysis (TNF-α, IL-10, and IL-1β) and protein related to pyrolysis signal pathway (NLRP3, ASC, caspase-1, GSDMD, and GSDME).

**Conclusions:**

Therefore, we speculate that HIF-1α promotes the proliferation and invasion of glial cells by regulating pyrolysis pathway. These results might provide a novel strategy and target for treatment of glioma.

## Background

Brain glioma is the primary malignant tumor with the highest incidence rate of the central nervous system in adults, with an annual incidence rate of about 6.4 per 100,000, of which the incidence rate of World Health Organization (WHO) grade 4 glioblastoma (GBM) is the highest, about 4.03/100,000, accounting for 50.1% of all primary malignant tumors of the central nervous system [1]. Although there are currently various treatment options, including surgical resection, radiotherapy, and chemotherapy, the survival period of patients is still relatively short. Research shows that the median overall survival (mOS) of patients with GBM after diagnosis is less than 1 year; after the STUPP front-line treatment regimen, the mOS of GBM patients can be increased to 16 months, while after the STUPP regimen combined with tumor treating field (TTF) treatment, the patient’s mOS can be extended to 20.9 months. However, the recurrence rate of GBM is close to 100%, and the treatment cost is high, which has caused great burden and economic pressure on patients, hospitals, and society [2, 3]. In recent years, with the deepening of research on the basic and clinical characteristics of glioma, the field of glioma has also been further developed; more and more biomarkers and potential treatment targets for glioma were proven to play important roles in disease progression, such as isocitrate dehydrogenase I (IDH) mutation, 1p/19q co-deletion, TP53 mutation, telomerase reverse transcriptase (TERT) promoter mutation, h3k27m mutation, and other key molecules; these molecular information are gradually used as pathological diagnosis [4]. Recent studies have shown that hypoxia inducible factor-1α (HIF-1α) were overexpressed in high-grade glioma tissues and were significantly associated with poor survival [5].

HIF-1 is a heterodimer, mainly composed of 120 kD HIF-1α and 91~94 kD HIF-1β [6]. Studies have shown that HIF-1α is overexpressed in gliomas and positively correlated with the degree of malignancy [7]. The expression of HIF-1α in high-grade gliomas was stronger than that in low-grade gliomas [8]. IDH1 gene mutation can activate HIF-1α signaling pathway [9]. IDH1 is one of the key enzymes of tricarboxylic acid cycle, and its coding gene mutation has a high frequency in gliomas, with glioma specificity [10]. Upregulation of HIF-1α can induce apoptosis [7], and recent studies have found that apoptosis can promote the occurrence and development of early tumors [11]. Pyroptosis, also known as cellular inflammatory necrosis, is a type of caspase-1-dependent programmed cell death that mediated by NOD-like receptor thermal protein domain associated protein 3 (NLRP3) inflammatory vesicles [12, 13]. The release of large amounts of inflammatory cytokines after the onset of pyroptosis can also promote malignant progression such as invasion and migration of glioma cells [14]. As seen from the above studies, the current glioma research mainly focuses on the effects of HIF-1α on glioma or pyroptosis on glioma, while the mechanism of whether HIF-1α plays important role on glioma through pyroptosis remains unclear.

A previous study by our group demonstrated that ouabain inhibited the survival of U-87MG cells and caused a dose-dependent reduction in cell viability, as well as inhibited HIF-1α expression [15]. Consistent with the findings of Shen et al. [16], silencing of the HIF-1α gene significantly inhibited the proliferation, invasion, and metastatic ability of U87 cells; in malignant astrocytomas, HIF-1α was mainly present in cells arranged in a pseudo-fenestrated pattern near the necrotic zone and in cells infiltrating normal tissue around the tumor [17]. HIF-1α protein was expressed in the nuclei of most glioblastoma cells, especially around the necrotic zone of glioblastoma [18]. Whether HIF-1α promotes the malignant evolution of glioma through the pyroptosis program remains to be further investigated. In the present study, we found for the first time that HIF-1α overexpression promotes the malignant evolution of human glioma cells through the pyroptosis-associated signaling pathway, which is reported below.

## Methods

### Clinical samples

The study was approved by the review board of the First Central Clinical School of Tianjin Medical University and conducted according to the principles of the Declaration of Helsinki. A total of 20 glioma tissues were collected at the department of neurosurgery. All tissues were stored at −80 °C for further studies. Written informed consent was obtained from each patient. Tissues were obtained during surgery and all cases were confirmed by pathologic diagnosis.

### Materials

Normal human brain cells and glioma cell lines (U251, SHG-44, U87) cells were purchased from Shanghai CAS cell bank; HIF-1α overexpressing adenovirus expression vector AAVrh.10 HIF-1α with adenovirus capsid AAVrh.10 Null was purchased from Jinan Sike Biotechnology Co. Apoptosis-associated speck-like protein containing caspase-recruitment domain (ASC), caspase-1, gasdermin-D (GSDMD), gasdermin-E (GSDME), and transforming growth factor β1 (TGF-β1) antibodies were purchased from Abcam. MTT, Trizol, and de-RNAase treated items were purchased from Sigma; RT-PCR primers were synthesized by Suzhou Jin Wei Zhi Biotechnology Co. DMEM medium, fetal bovine serum, trypsin, and other cell culture reagents were purchased from Gibco, USA, and the BCA protein kit was purchased from Biyuntian Technology Co.

### Cell culture and transfection

The experiment was divided into 3 groups, blank control group (sham group), negative control group (Null group), and overexpression group (HIF-1α group). SHG44 cells in the Null and HIF-1α groups were added with equal titers of purified adenovirus expression vector AAVrh.10 Null and AAVrh.10 HIF-1α, respectively. The sham group was added with equal volume of PBS and incubated for a total of 12 h. The cells were changed or passaged according to the density of the cells. At each passaging, 2.5 µg/mL puromycin was added to continue screening for stable cell lines, and cell GFP fluorescence intensity was observed at fluorescence microscopy every other day until there was no significant change in fluorescence intensity and no dead floating cells. A portion of the cells were used for subsequent RT-PCR, transwell, western blot, and other experiments. The other part was frozen and stored.

### Real-time PCR for HIF-1α mRNA expression

RNA from brain tissues and glioma cell lines cells were extracted using Trizol and reverse transcribed into cDNA strands using a reverse transcription kit. The expression of HIF-1α mNRA was detected using a fluorescent quantitative PCR kit. β-Actin was used as an internal reference for HIF-1α. The primer sequences were as follows: HIF-1α, forward: 5′AATAAGTGGT GGTTACTCAG3′; reverse: 5′AAATAAACAT CTCTGTGGA CCAGG3′.

### MTT staining assay

The cells of each group in the logarithmic growth phase were counted and adjusted to a cell concentration of 2 × 105 cells/mL with 1640 DMEM complete medium, inoculated in 96-well plates with 50 µL per well, and three replicate wells were set up for each group. After 12-h incubation, 20 µL of MTT solution was added to each well, and incubation was continued for 4 h, 150 µL of DMSO was added to terminate the reaction, 490 nm wavelength was selected, and the light absorption value of each well was measured on an ELISA monitor, and the results were recorded for 48 h, and the cell growth curve was plotted with time as the horizontal coordinate and absorbance value as the vertical coordinate.

### Cell clone formation assay

The cell suspension was made from each group of cells at logarithmic growth stage. The cell suspensions were diluted in a gradient multiple, inoculated in dishes containing culture medium, and cultured for 2–3 weeks. The culture was terminated when clones were visible to the naked eye in the dishes. The supernatant was discarded, fixed in 4% paraformaldehyde, and stained with GIMSA staining solution for 10–30 min. Photographs were taken and counted. Calculate the clone formation rate, clone formation rate = (number of clones/number of inoculated cells) × 100%.

### Transwell assay

Use serum-free DMEM medium to resuspend each group of cells to 1 × 10^4^/mL, take 200 µL and add it into transwell chambers, and place the transwell chambers with cell suspension in the experimental wells containing culture medium for 24 h. Remove the transwell chambers, wash them with PBS, and then wipe off the membrane of the chambers with cotton swabs. The cells on the upper layer of the membrane of the chambers were wiped off using cotton swabs, and the cells on the lower layer of the membrane were stained with crystal violet and placed under a microscope for counting and photographing.

### Cell scratch assay

The cells of each group in the logarithmic growth phase were counted and inoculated in 6-well plates, 5 × 10^4^ cells/well, with 2 replicate wells for each group. After the cells were adhered to the wall, the bottom of the culture was scratched with a pipette tip in the shape of “|,” and the culture medium was heated after PBS rinsing and continued to be incubated for 24 h. The migration of cells in the scratched area was observed and photographed.

### Western blot

Cells from each group at 72 h post-transfection were taken, crushed and proteins were extracted, and the total protein amount of each sample was measured using the BCA protein assay kit. Equal amounts of 50 µg of protein were extracted from each group, and the proteins were separated using sodium dodecyl sulfate–polyacrylamide gel electrophoresis and transferred semi-dry to PVDF membranes. The membranes were closed with TBST buffered saline containing 10% skim milk powder for 1 h at 4 °C, and then overnight in TBST buffer containing primary antibodies: rabbit monoclonal anti-NLRP3 antibody (1:1000), rabbit monoclonal anti-ASC antibody (1:1000), rabbit monoclonal anti-caspase-1 antibody (1:1000), rabbit monoclonal anti-GSDMD antibody (1:1000), rabbit monoclonal anti-GSDME antibody (1:1000), rabbit monoclonal anti-TGF-β1 antibody (1:1000), TBST was rinsed 4 times, and then the membranes were incubated with horseradish peroxidase goat anti-rabbit (1:10,000) secondary antibody for 2 h. TBST was rinsed 4 times, and ECL plus was used for signal detection, and Image J software was used for quantitative analysis.

### ELISA assay

The cells of each group at 72 h after transfection were taken, crushed and protein extracted, and the total protein amount of each sample was measured using the BCA protein assay kit. The expression of TNF-α, IL-10, IL-17, and IL-1β was measured according to the instructions of the ELISA kit.

### Immunofluorescence assay

Crawled cell-filled slides were fixed with 4% paraformaldehyde for 15 min, washed with PBS, permeabilized with 0.5% Triton X-100 (PBS) at room temperature for 20 min, washed with PBS, and closed with goat serum at room temperature for 30 min; primary antibodies (NLRP3, caspase-1, β-actin) (1:1000) were incubated overnight at 4 °C; the sections were subsequently rinsed with PBS and incubated with secondary antibodies (NLRP3: red donkey anti-rabbit IgG red fluorophore, 1:1000; caspase-1: green donkey anti-rabbit IgG green fluorophore, 1:1000) for 1 h at room temperature, respectively. Final staining was done using DAPI. All sections were viewed under confocal fiberscope. The area of positive fluorescence was quantified by computational scanning software.

### Prognostic value assessment

Download the dataset mRNAseq_325 from the Chinese glioma genome atlas (CGGA) database, which contains clinical data and mRNA sequencing data of glioma patients. Extract the expression level of HIF-α gene and match it with clinical data, and remove some cases with missing clinical data. Compare the differences in the expression level of HIF-α in different grades of glioma. Subsequently, select 222 patients with primary glioma, and divide the selected cases into high-expression group (111 cases) and low-expression group (111 cases) based on the median value of HIF-α expression in glioma tissues, and plot the survival curves of the two groups.

### Statistical analysis

SPSS17.0 software was used to process the experimental data, GraphPad Prism 5 software was used for plotting, western blot and immunofluorescence data were expressed as mean ± standard deviation $\left(\overline{x} \pm \mathrm{s }\right)$, *t*-test was performed for two-by-two comparison, and one-way ANOVA was used for comparison between groups; *P* < 0.05 and *P* < 0.05 were considered a statistically significant difference.

## Results

### HIF-1α gene overexpression promotes HIF-1α mRNA and protein expression in glioma SHG44 cells and alters the morphology of glioma SHG44 cells

**Fig. 1:**
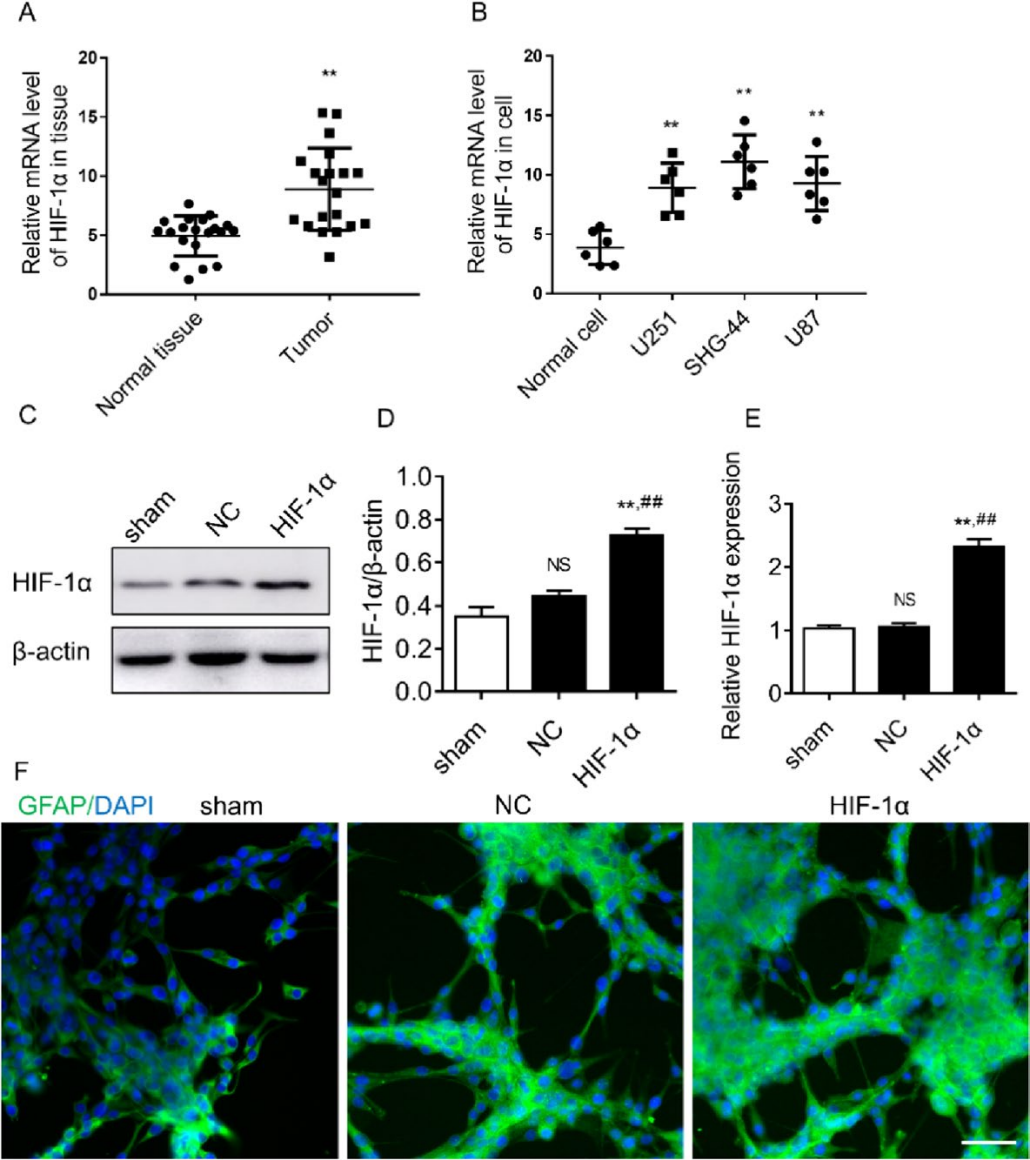
The expression of HIF-1α and morphology of glioma cells. **A** Expression levels of HIF-1α mRNA in brain tissue. **B** Expression levels of HIF-1α mRNA in cell lines. **C** HIF-1α mRNA expression in each group. **D** Immunoblot bands of HIF-1α and β-actin protein. **E** Relative expression of HIF-1α protein. **F** Morphology of glioma cells in each group under immunofluorescence, GFAP in green, DIPI in blue, scale bar is 40×. ^NS^*P* > 0.05 compared with sham group, **P < 0.01, ^##^*P* < 0.01 compared with NC group

To explore the role of HIF-1α in glioma development, we first examined the mRNA expression of HIF-1α in glioma tissues and glioma cells. The mRNA expression level of HIF-1α was significantly elevated in glioma tissue compared with adjacent normal tissues (*P *< 0.01, Fig. 1A); HIF-1α mRNA expression was significantly elevated in U251, SHG-44, and U87 glioma cell lines when compared with normal brain cells (*P* < 0.01, Fig. 1B). Compared with U251 cells and U87 cells, SHG-44 cells had the highest expression levels of HIF-1α mRNA without statistical significance. These data suggest that HIF-1α may be involved in the development and progression of glioma. The expression of HIF-1α mRNA and protein were significantly higher in the HIF-1α group compared with the sham group, and the difference was statistically significant (*P* < 0.05); compared with the NC group, the expression of HIF-1α mRNA and protein were significantly higher in the HIF-1α group, and the difference was statistically significant (*P* < 0.05) (Fig. 1C–E). Glioma cells in the NC group were slightly enlarged in size under light microscopy compared with the sham group, while glioma cells in the HIF-1α group were significantly swollen and expanded with many bubble-like protrusions compared with the NC group (Fig. 1F).

### HIF-1α overexpression promotes glioma cell proliferation, invasion, and expression of inflammatory cytokines

**Fig. 2:**
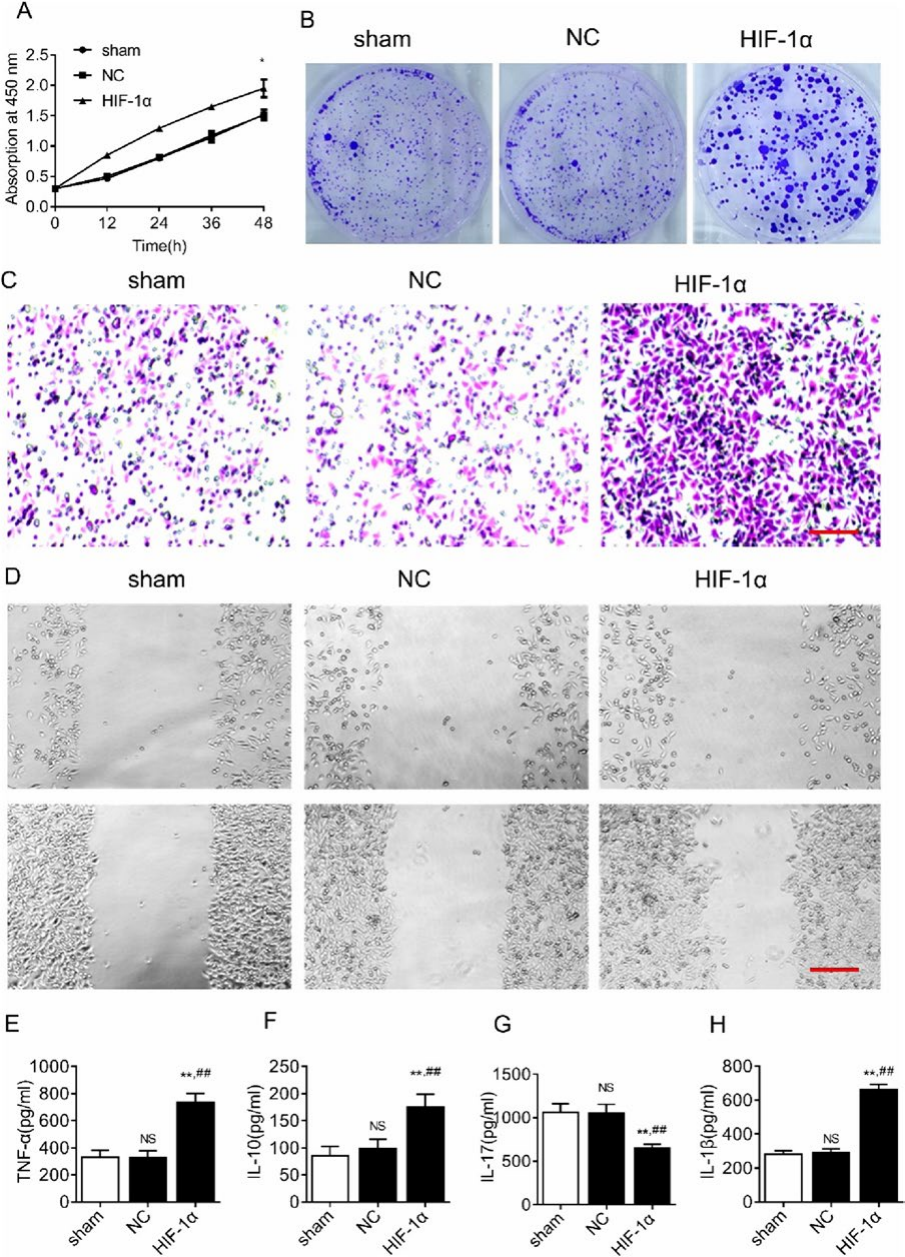
HIF-1α overexpression affects proliferation, invasion, and inflammatory cytokines of SHG44 cells. **A** MTT cell proliferation assay. **B** Cell cloning assay. **C** Transwell assay, scale bar is 20×. **D** Cell scratch assay, scale bar is 10×. **E** TNF-α expression. **F** IL-10 expression. **G** IL-17 expression. **H** IL-1β expression. ^NS^*P* > 0.05, *P < 0.05, **P < 0.01 compared with sham group and ^##^*P* < 0.01 compared with NC group

The MTT results showed that there was no difference in the cell proliferation rate between the sham and NC groups (*P* > 0.05), and the cell proliferation rate was significantly higher in the HIF-1α group compared with the sham and NC groups, and the differences were statistically significant (*P* < 0.05) (Fig. 2A). The results of cell cloning assay showed that the HIF-1α group was able to significantly promote the clonogenic ability of cells and promote glioma cell stemness compared with the sham and NC groups (Fig. 2B). Transwell assay results showed that there was no difference in the number of membrane penetrating cells [(139.7 ± 12.5) vs (140.5 ± 10.2)] between the sham and NC groups (*P* > 0.05), and the number of perforated cells was significantly higher in the HIF-1α group (326.4 ± 15.6) compared with the sham and NC groups (*P* < 0.05) (Fig. 2C). The results of cell scratching showed that there was no difference in cell migration rate between the sham and NC groups, and the cell migration rate was significantly higher in the HIF-1α group compared to the sham and NC groups (Fig. 2D). The results of ELISA experiments showed that there was no significant difference in the expression levels of pyroptosis-related inflammatory factors (TNF-α, IL-10, IL-17, and IL-1β) between the sham and NC groups (*P* > 0.05); the expression levels of pyroptosis-related inflammatory factors (TNF-α, IL-10, and IL-1β) were significantly higher in the HIF-1α group compared with both the sham and NC groups (*P* < 0.05), while the expression level of tumor suppressor IL-17 was nominally decreased (*P* < 0.05) (Fig. 2E–H).

### The effect of HIF‐1α on pyroptosis markers

**Fig. 3:**
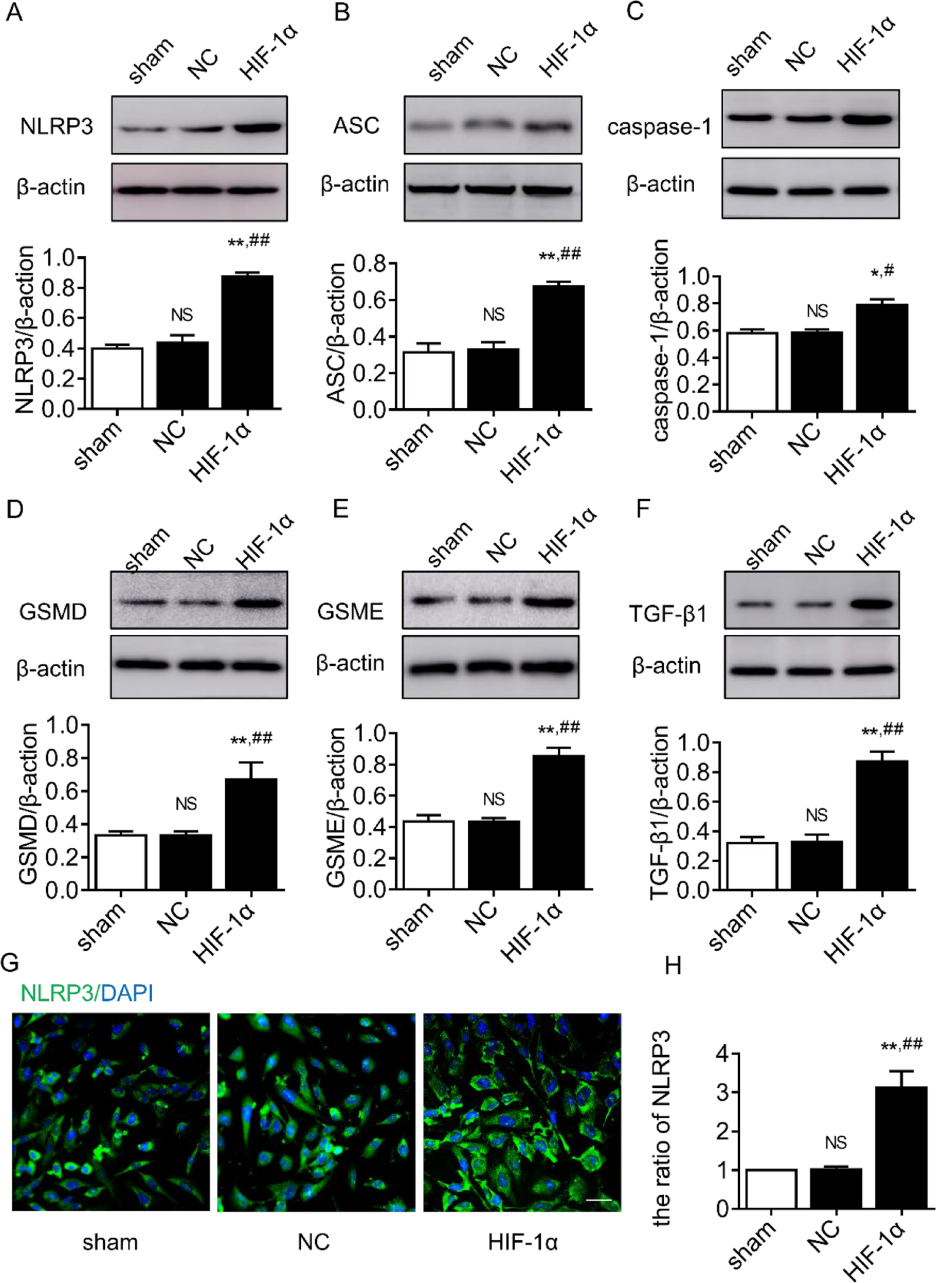
The effect of *HIF*‐*1α* on pyroptosis markers. **A** NLRP3 protein expression. **B** ASC protein expression. **C** Caspase-1 protein expression. **D** GSDMD protein expression. **E** GSDME protein expression. **F** TGF-β1 protein expression. **G** Green-labeled NLRP3 and blue-labeled DAPI. **H** NLRP3 fluorescence quantification; ^NS^*P* > 0.05, **P < 0.01 compared to sham group, ^##^*P* < 0.01 compared to NC group, scale bar 40×

The results of western blot experiments showed that there was no significant difference in the expression levels of pyroptosis-related proteins (NLRP3, ASC, caspase-1, GSDMD, GSDME, and TGF-β1) between the sham and NC groups (*P* > 0.05); the expression levels of pyroptosis-related proteins (NLRP3, ASC, caspase-1, GSDMD, GSDME, and TGF-β1) were significantly higher in the HIF-1α group compared with both the sham and NC groups (*P* < 0.05) (Fig. 3A–F). Immunofluorescence results showed no significant difference in NLRP3 expression between the sham and NC groups (*P* > 0.05), and NLRP3 expression levels were significantly higher in the HIF-1α group compared to both the sham and NC groups (*P* < 0.05) (Fig. 3G and H).

### The role of HIF-α in prognosis evaluation

**Fig. 4:**
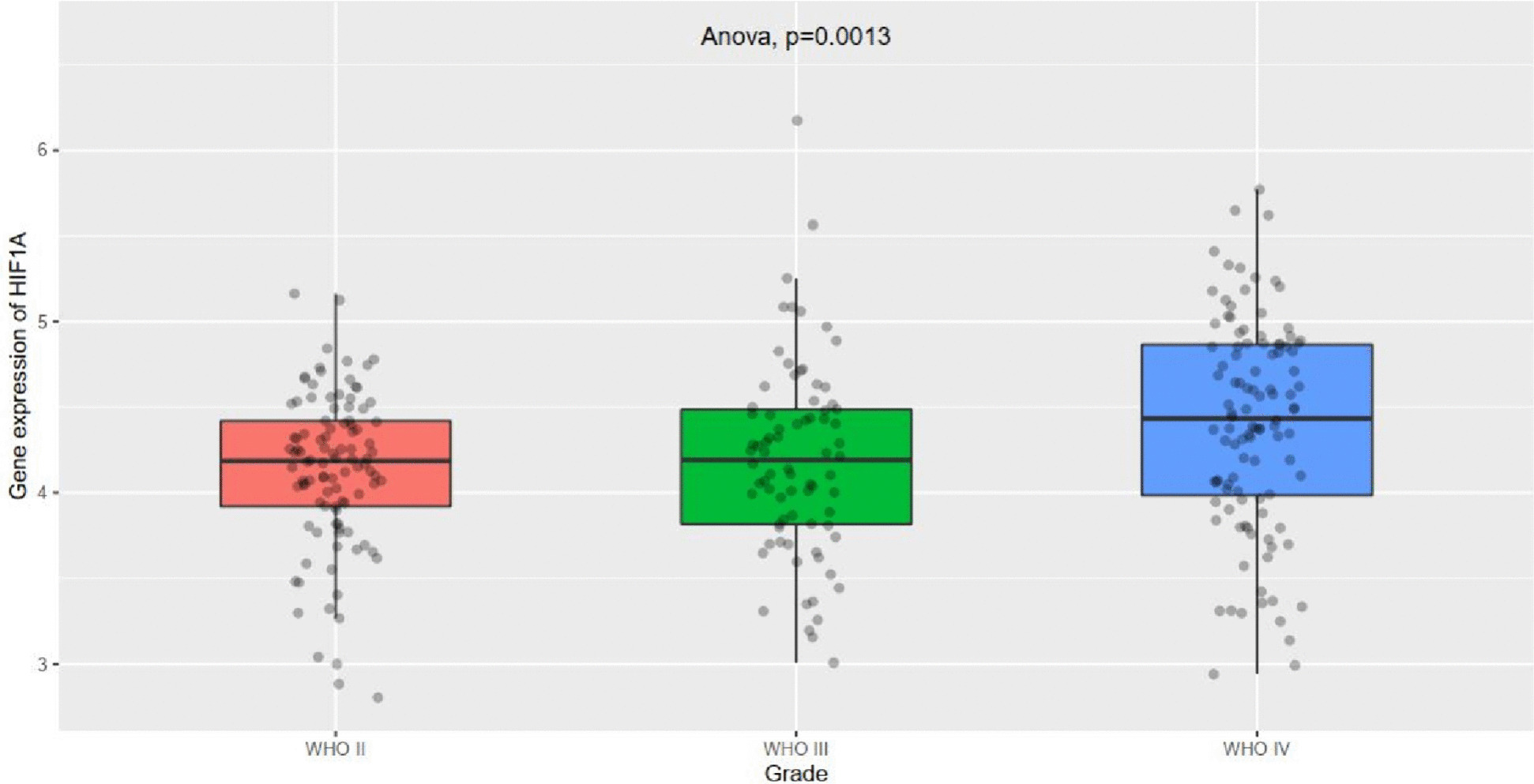
The expression of HIF-α in different grades of gliomas

**Fig. 5:**
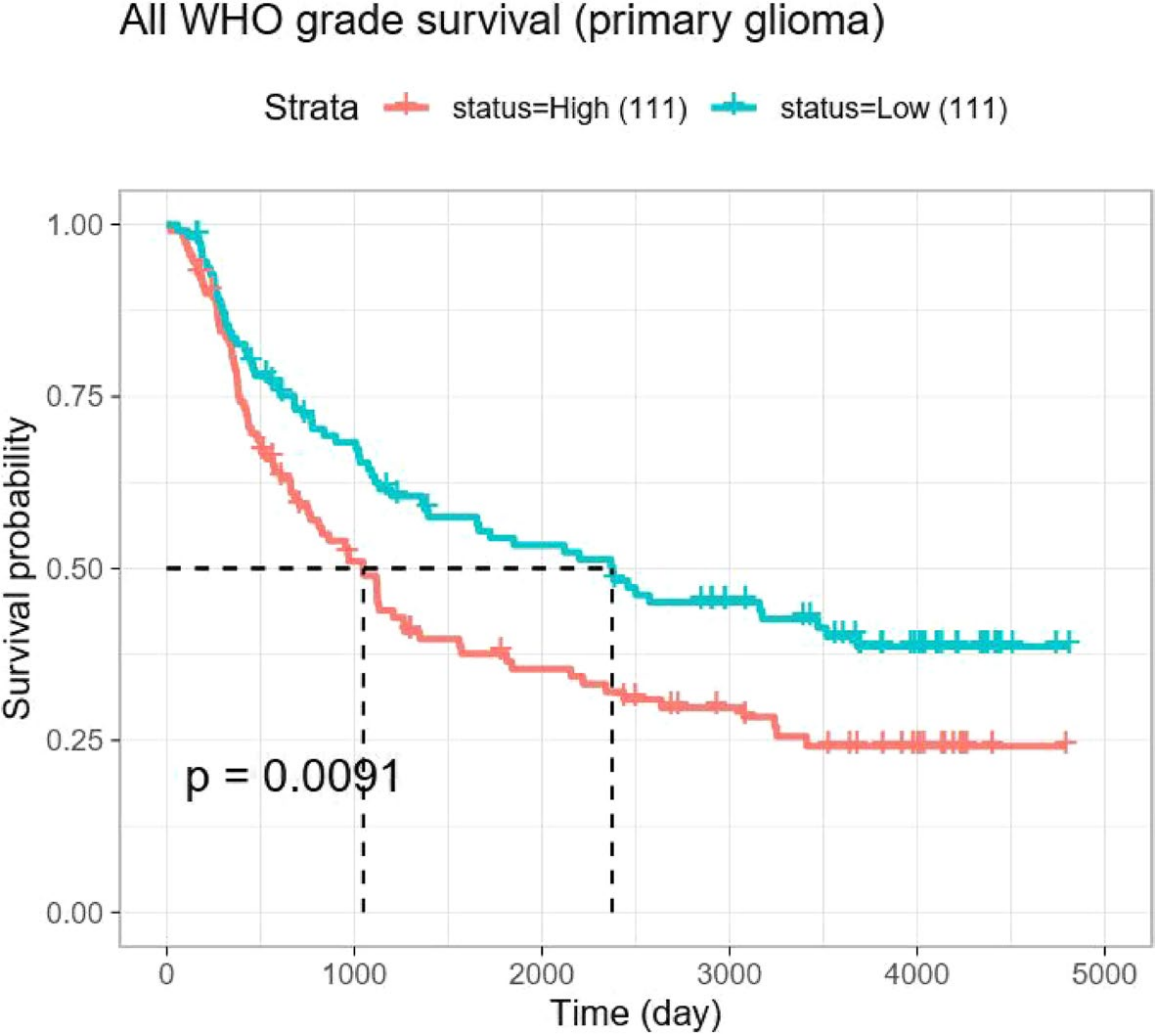
Comparison of survival curves between the two groups

The expression of HIF-α showed significant differences among different grades of gliomas, with higher levels of HIF-α expression in grade IV gliomas compared with grade II or III gliomas (*P* < 0.05) (Fig. 4). Compared with the low-expression group of HIF-α, the median survival time of the high-expression group was shorter, and the difference was statistically significant (*P* < 0.05) (Fig. 5).

## Discussion

Tumors arise from gene mutations, and IDH1 mutations are glioma-specific, and the mutations are capable of causing HIF-1α overexpression, which may be important for the development of gliomas [10]. This study found that when glioma cells overexpressed the HIF-1α gene, the cell’s proliferation and invasion abilities significantly increased, as well as the cell’s stemness. This suggests that overexpression of the HIF-1α gene may promote the increase in the malignant degree of glioma. Recent studies show that HIF-1α, a novel oncogene, is closely associated with the development and progression of glioma and predicts patient prognosis [19]. HIF-1α expression is significantly elevated in gliomas and positively correlates with malignancy [11]. In malignant astrocytomas, HIF-1α was mainly present in cells arranged in a pseudo-fenestrated pattern near the necrotic zone and in cells infiltrating normal tissue around the tumor. HIF-α protein is expressed in the nuclei of most glioblastoma and mesenchymal astrocytoma cells and is particularly highly expressed around the necrotic zone of glioblastoma [20]. Through the clinical data validation of glioma patients in the CGGA database, it was also confirmed that the overexpression of HIF-1α gene was associated with poor long-term prognosis. Further detection of pyroptosis-related inflammatory factors revealed that the overexpression of HIF-1α could promote the increase of inflammatory factors, thereby promoting the formation of an inflammatory environment. Tumorigenesis and progression cannot be separated from the tumor microenvironment [21]. The inflammatory microenvironment, suppressive immune microenvironment, migratory microenvironment, and angiogenesis constitute a tumor microenvironment with low oxygen, low pH, and high pressure [22]. Several cytokines, including TNF-α, IL-10, IL-17, and IL-1β, have been shown to play an important role in the tumor inflammatory environment or tumor immune response [23]. TNF-α can be secreted by tumor cells and host cells in the tumor microenvironment to enhance the oncogenic process [24]. Compared to TNF-α, IL-10 and IL-17 play a more direct role in the mechanism of tumor immune escape. IL-10 receptor-deficient mice are more prone to developing colon cancer, and IL-10 receptor-deficient persons are more likely to suffer malignant B-cell lymphomas at a young age [25]. IL-17 is secreted by Th17 cells; it can trigger antitumor responses and lead to tumor shrinkage through immune addition [26, 27]. TGF-β1 is an important molecule for differentiation of Treg and Th17 cells and can be induced by HIF-1α expression [28]. Altered expression of TGF-β1 can regulate Th17/Treg cell homeostasis, which in turn affects tumor survival [29]. In addition, IL-1β-mediated inflammatory vesicles can be involved in the process of caspase-1-related cell pyroptosis and connects the complex relationship between carcinogenesis and inflammation-induced cell pyroptosis [30].

This study found that overexpression of HIF-1α promotes the expression of cell pyroptosis-related proteins in glioma cells. Cell pyroptosis, as a programmed death, is morphologically characterized by both apoptosis and necrosis [31]. It has recently been shown that cell pyroptosis promotes early tumorigenesis [32]. This is mainly due to two reasons: first, JNK kinase is activated and translocated to the nucleus in response to cytotoxin secreted by tumor cells, which promotes the expression of pyroptosis-related genes and initiates the pyroptosis program to control tumor development; second, the inflammatory environment caused by tumors can promote the formation of inflammatory vesicles NLRP3, which promotes the formation of GSDMD by activating caspase-1 and increases the secretion of the inflammatory cytokines IL-1β and IL-18 from cell membrane pores, which leads to inflammation-associated pyrophosphorylated cell death [33, 34]. In addition, other studies have found that cellular pyrophosphorylation also in turn promotes NLRP3 inflammatory vesicle activation, thereby promoting tumor cell proliferation and metastasis [35, 36].

## Conclusion

This study provides evidence that HIF-lα promotes the proliferation and invasion of glial cells by regulating pyrolysis pathway. These results might provide a novel strategy and target for treatment of glioma.

## Data Availability

Please contact author for data requests.
